# Nanoengineered thermoresponsive magnetic hydrogels for biomedical applications

**DOI:** 10.1002/btm2.10034

**Published:** 2016-10-17

**Authors:** Nima A. Jalili, Madyson Muscarello, Akhilesh K. Gaharwar

**Affiliations:** ^1^ Dept. of Biomedical Engineering Texas A&M University, College Station TX 77843; ^2^ Dept. of Materials Science and Engineering Texas A&M University, College Station TX 77843; ^3^ Center for Remote Health Technologies and Systems, Texas A&M University, College Station TX 77843

**Keywords:** hydrogels, magnetic nanoparticles, nanocomposites, thermoresponsive, tissue engineering

## Abstract

“Smart” hydrogels are part of an emerging class of biomaterials that respond to multiple external stimuli. A range of thermoresponsive magnetic hydrogels is currently being developed for on‐demand delivery of biomolecules for a range of biomedical applications, including therapeutic drug delivery, bioimaging, and regenerative engineering. In this review article, we explore different types of magnetic nanoparticles and thermoresponsive polymers used to fabricate these smart nanoengineered hydrogels. We highlight some of the emerging applications of these stimuli‐responsive hydrogels for biomedical applications. Finally, we capture the growing trend of these smart nanoengineered hydrogels and will identify promising new research directions.

## 1 Introduction

Stimuli‐responsive hydrogels can be designed to respond to changes in external stimuli, such as temperature, pH, light, and ultrasonic frequency.^1^, ^2^, ^3^, ^4^, ^5^ Hydrogels that experience physio‐chemical changes due to a change in an external stimulus have great potential in the field of noninvasive and remote controlled therapies.^5^, ^6^, ^7^, ^8^, ^9^, ^10^ However, the use of these external stimuli (temperature, pH, light, and ultrasound) limits the applications of these responsive hydrogels systems to specific tissue regions. For example, thermo‐responsive hydrogels can only be used for skin‐deep penetration, where external heating can be applied, while pH‐responsive hydrogels can only be used in tissue with specific pH ranges.^4^, ^11^, ^12^ Hydrogels responding to ultrasound waves and near‐IR radiation have limited penetration ability due to high diffraction and the absorption ability of surrounding tissues. This problem of tissue‐specific stimuli can be circumvented through the use of hydrogels containing nanoparticles.^13^, ^14^, ^15^

A range of nanoparticles are incorporated within polymeric hydrogels to mechanically reinforce the hydrogel network and/or obtain desired stimuli‐response. For example, two‐dimensional (2D) graphene nanosheets^16^, ^17^ or nanosilicates^18^, ^19^, ^20^ can be incorporated within polymeric hydrogels for sustained and controlled release of therapeutic and to mechanically reinforce the network. In addition, nanoparticles can physically or chemically interacts with the hydrogel network and can results nanocomposite network with unique property combinations.^21^, ^22^ Surface decorated nanoparticles can covalently interact with polymeric chains and can mechanically reinforce the network at ultralow concentration.^23^ Developing nanoengineered hydrogels with tailored characteristics and functionality has opened up new possibilities in developing advanced biomaterials for various biomedical applications.^24^, ^25^

Magnetic nanoparticles (MNPs), in response to a generalized external stimulus, can internally stimulate changes to the polymeric network of the hydrogel, allowing for a versatile stimuli‐responsive system.^13^, ^14^, ^15^ MNPs respond to an alternating magnetic field (AMF) and have been shown to generate heat due to their superparamagnetic nature.^26^, ^27^, ^28^, ^29^, ^30^, ^31^, ^32^, ^33^, ^34^ Due to this characteristic, MNPs are extensively used as magnetic contrast agents to improve imaging resolution for magnetic resonance imaging (MRI) as well as hyperthermia treatments.^26^, ^29^, ^35^ By combining these MNPs with thermoresponsive polymers, a safe, deep‐tissue response can be elicited to control the material behavior of the “smart hydrogel.” These thermoresponsive magnetic nanomaterials can be designed for a wide array of biomedical applications; such responsive materials can be utilized for delivering therapeutics to tumor sites through leaky vasculature, while others are designed to provide high contrast during MRI scanning.^36^, ^37^, ^38^, ^39^, ^40^

**Figure 1:**
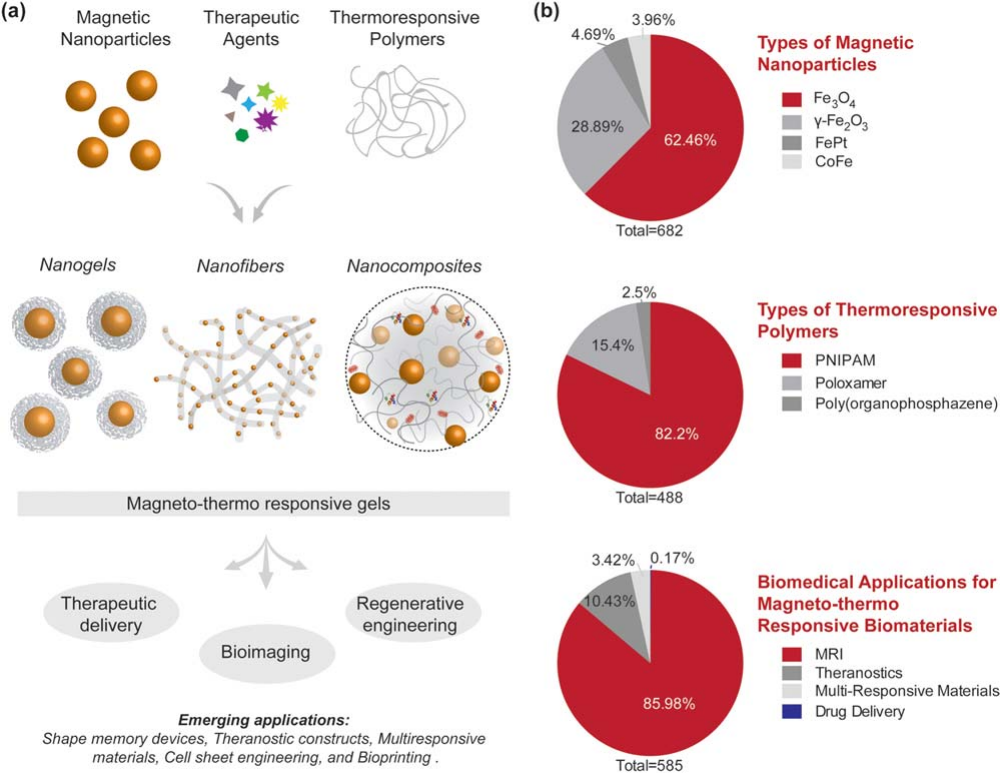
Magnetically responsive nanoengineered hydrogels for biomedical applications. (a) Schematic showing different types of nanomaterials fabricated from magnetic nanoparticles and thermoresponsive polymers. Common biomedical applications of magnetically‐responsive nanocomposites. (b) Pie charts showing distribution of different types of magnetic nanoparticles, and thermoresponsive polymers for various biomedical applications. Data obtained from ISI Web of Science in June 2016

In this review, we will specifically focus on nanoengineered thermoresponsive hydrogels loaded with magnetic nanoparticles. A range of articles is available on biomedical applications of thermoresponsive polymers^1^, ^2^, ^3^, ^4^, ^5^, ^6^, ^7^, ^8^, ^9^, ^10^ and magnetic nanoparticles.^26^, ^27^, ^28^, ^29^, ^30^, ^31^, ^32^, ^33^, ^34^ This review focuses on a subset of thermoresponsive hydrogels that respond to external magnetic fields. We will first introduce different types of magnetic nanoparticles and thermoresponsive polymers that are used to design thermoresponsive magnetic hydrogels (Figure 1a). We will critically evaluate and discuss biomedical applications—including therapeutic delivery, bioimaging and regenerative engineering of these composite hydrogels. Finally, we will capture the growing trend of these smart nanoengineered hydrogels and will identify promising new research directions.

## 2 Magnetic nanoparticles and thermoresponsive polymers used to fabricate smart hydrogels

### 2.1 Magnetic nanoparticles

Among the different types of MNPs, ferromagnetic, and superparamagnetic nanoparticles have been extensively investigated for biomedical applications (Figure 1b).^29^, ^33^, ^41^, ^42^, ^43^, ^44^ Superparamagnetic MNPs are less than 30 nm in diameter and under an AMF these MNPs produce heat due to Brownian and Néel relaxation.^32^ This localized heat response can be used to ablate cells.^35^ However, due to their small size and large surface area, these MNPs may aggregate and thereby lose superparamagnetic characteristics.^26^ To improve the stability of MNPs and prevent aggregation, the nanoparticle surface can be functionalized with various small molecules including poly(ethylene glycol) (PEG), citric acid, and oleic acid.^30^ Magnetite and hematite are the most commonly utilized ferromagnetic nanoparticles for biomedical applications due to their biocompatibility, physiological stability, and high magnetization ability. Magnetite, which has the chemical formula Fe_3_O_4_ and may also be written as Fe_2_O_3_⋅FeO, contains Fe^3+^ as well as Fe^2+^ ions ordered unequally, which gives rise to a net magnetization ability, as well as superparamagnetic capabilities. This high magnetization ability makes magnetite a prime agent for use as an MRI agent, hyperthermic agent, and a component in drug delivery constructs. Magnetite can also be used in the creation of a scaffold‐free tissue culture.^26^, ^30^, ^45^, ^46^ The other common ferromagnetic nanoparticle, hematite, has the formula Fe_2_O_3_ and can be functionalized with fullerenes such as C_60_ for potential use in drug delivery, nonviral gene delivery, and MRI contrast agents.^47^, ^48^, ^49^ Another iron‐containing nanoparticle used in biomedical applications is iron platinum (FePt). The modified surface of FePt nanoparticles allows for applications in targeted drug delivery, photochemical therapy, biosensing, and imaging.^42^, ^50^, ^51^ Finally, cobalt‐iron (CoFe) nanoparticles are also under investigation for potential bioimaging and drug delivery applications.^41^, ^52^, ^53^ Overall, superparamagnetic iron‐containing nanoparticles (SPINs) are utilized in research for a wide range of biomedical applications and have potential for clinical translation due to low cell toxicity.^54^, ^55^

MNPs are biocompatible and biodegradability and thus have been investigated for a range of biomedical applications. MNPs are used in clinical diagnostics (MRI contrast agents [Feridex I.V. ®]) and treatment (liposomal drug delivery systems for cancer therapy [Doxil®]). MNPs can be metabolized and releases iron ions, which can be stored in body and eventually used by erythrocytes to form hemoglobin. However, biocompatibility of thermoresponsive magnetic hydrogels will depend on various parameters such as type of polymeric network and structures of MNPs. In addition, the shape, size and surface coating of MNPs will directly control metabolism, clearance, and toxicity profiles of MNPs. Therefore, additional investigation on long‐term biocompatibly, biodistribution, and clearance need to be investigated in detail.

### 2.2 Thermoresponsive polymers

Thermoresponsive hydrogels exhibit phase transition behavior from hydrophilic to hydrophobic states due to a change in temperature.^1^, ^2^, ^3^, ^4^, ^5^, ^6^, ^7^, ^8^, ^9^, ^10^ By using the Néel and Brownian relaxation mechanisms of magnetic nanoparticles, which can be either suspended or encapsulated within these thermoresponsive hydrogels, the swelling and de‐swelling behavior of the polymeric network can be controlled by external magnetic fields. A range of synthetic polymers including poly(*N*‐isopropylacrylamide) (PNIPAM), poloxamer, and poly(organophosphazene) (PPZN) have been utilized in thermoresponsive hydrogels for biomedical applications (Figure 1b).

PNIPAM, has been extensively investigated with potential applications in drug delivery, cell sheet engineering, tissue engineered scaffolds, and DNA sensing.^12^, ^56^, ^57^, ^58^, ^59^, ^60^ PNIPAM has its lower critical solution temperature (LCST) at 32 °C, and can be copolymerized with hydrophilic or hydrophobic polymers to increase or decrease the transition temperature, respectively. The process of hydrophilic to hydrophobic transition at LCST is reversible, which can be designed to load and deliver therapeutics. Poloxamer is the generic name for tri‐block copolymers consisting of two blocks of poly(ethylene oxide) (PEO) and a block of poly(propylene oxide) (PPO).^4^ Poloxamers are sold commercially as Pluronics® and have exhibited modular LCST depending on molecular weight and the ratio between PEO and PPO segments. PPZN is a family of macromolecules first isolated in the 1960s; they have been found to exhibit a reversible sol‐gel transition with an associated change in temperature.^61^

Thermoresponsive natural polymers such as polypeptides and artificial poly(amino acid)s have shown LCST‐like behavior.^11^, ^62^ For example, natural elastin consist of Val‐Pro‐Gly‐Val‐Gly (Val = valine, Pro = proline, Gly = glycine) (L46), demonstrate a transition temperature ∼27 °C. Similarly, elastin‐like polypeptides and collagen‐mimetic peptides, can undergo a thermally reversible phase transition to form mechanically resilient hydrogels. However, very limited studies have investigated thermoresponsive natural polymers loaded with MNPs for biomedical applications.

## 3 Magnetically triggered thermo‐responsive nanogels and nanocomposites for drug delivery

Optimizing doses and rate of therapeutic delivery are important for a range of clinical procedures. Many analgesics utilize delayed release or diffusion kinetics to control the delivery of drugs within the therapeutic window. However, most of these approaches are administered systemically and due to limited control over therapeutic diffusion rate, effective concentration is difficult to sustain for a prolonged duration. In addition, many drugs are water insoluble and have a short half‐life. These factors, combined with the potentially serious side effects of systemic toxicity due to a dose above the therapeutic window, are partially responsible for many pharmaceuticals' harmful side effects. In order to reduce these side effects, while maintaining drug effectiveness, a range of polymeric and composite nanoparticles have been investigated as a potential drug delivery vehicle. A drug delivery vehicle small enough to enter into tissue can increase cellular uptake, as seen in smaller molecules that exist systemically, but in order to work effectively in vivo, the nanoparticles also have to be biocompatible. In order to achieve a controlled drug release, the immune system cannot interfere with the delivery vehicles. When applying nanocomposite systems for drug delivery or other applications, interference from the immune system will cause digestion of the construct by macrophages and cause either burst release or prevent release by fibrous encapsulation. Traditionally, thermoresponsive polymer encapsulating magnetic nanoparticles are designed for controlled drug delivery and a range of review articles highlight these approaches. In this section, we will highlight some of these approaches to overcome some of the above listed limitations.

**Figure 2:**
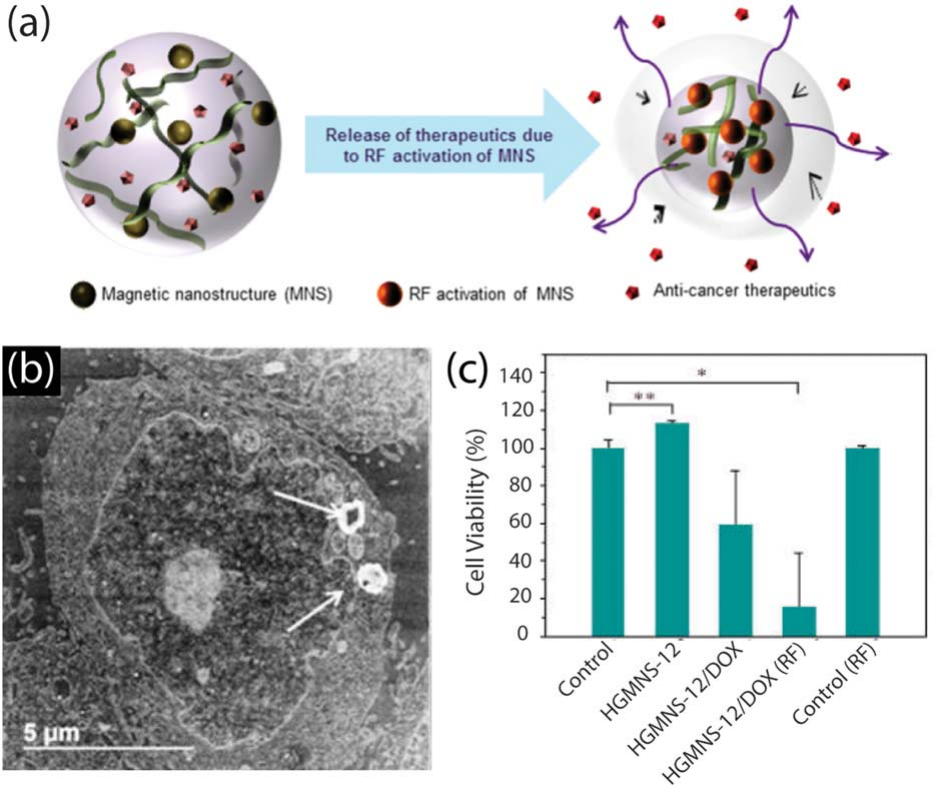
Stimuli responsive nanogels for drug delivery. (a) Schematic showing magnetically‐triggered hydrogel for on‐demand release of therapeutics. (b) Electron microscopy showing uptake of drug(DOX)‐loaded nanogels (HGMNS) by cancer cells (HeLa), identified by white arrows. (c) HeLa cell viability under different conditions demonstrating efficacy of magnetically triggered drug release from nanogels. Adapted and reproduced^63^ with permission from American Chemical Society © 2014

Recently, magneto‐thermoresponsive nanocomposites have been synthesized by conjugating Fe_3_O_4_ nanoparticles with either PEG or polyhedral oligomeric silsesquixone (Figure 2).^63^ The particles were then encapsulated by copolymerizing a layer of PNIPAM to produce a nanocomposite hydrogel system for in vitro release of incorporated Doxorubicin with and without presence of an AMF field. At temperatures above the material's intrinsic lower critical solution temperature (LCST), the PNIPAM expels its water content to form globules and precipitates out of solution. This occurs because hydrophobic constituents (isopropyl) of the polymers forming the hydrogel become entropically favored over the hydrogen bonding from hydrophilic components, resulting in thermally induced conformational changes. They observed that a 48‐hr incubation period after 1 hr of AMF exposure allowed for the highest release of entrapped drug (∼40% or 10 µg of DOX) and resulted in <20% viability. This show the efficacy of nanogels for controlled release of therapeutics.

In another similar approach, thermoresponsive Pluronic® P85, was loaded with magnetic nanoparticles for drug delivery.^64^ Pluronic® P85, a block co‐polymer of PEO and PPO blocks, undergoes a hydrophilic to hydrophobic transition below its transition temperature. This behavior is known as a critical micellar temperature (CMT). They covalently conjugated functionalized magnetic nanoparticles to Pluronic® P85 via amino groups to obtain MagPluronics. The nanocomposite system was synthesized at a temperature lower than CMT to encapsulate curcumin. They showed a sustained release of curcumin of more than 4 days under physiological conditions. The proposed system demonstrates a thermoresponsive system where the magnetic nanoparticles acted as a nucleating agent for the covalently bound theremoresponsive polymer to nanocarriers for stimuli‐responsive drug delivery. Additional studies are required to show the theremoresponsive behavior of these nanoengineered system.

Synthetic and natural polymers can be combined to fabricate thermoresponsive magnetic hydrogels. A multiresponsive PNIPAM grafted carboxymethylchitosan (CMC) composite loaded with MNPs was fabricated.^65^ The PNIPAM was synthesized *via* free radical polymerization in the presence of CMC and surface functionalized MNP, and then crosslinked with glutaraldehyde to form water swellable microspheres. The microspheres were shown to be both temperature and pH responsive with a LCST of ∼50 °C and an activating pH of 11, respectively. The microspheres have a potential for drug delivery, releasing 87% of the model drug indomethacin after 48 hr above its LCST. Drug release is further facilitated through exposure to a basic pH (pH = 11), and is inhibited upon exposure to acidic pHs.

Another approach to design nanocomposite constructs is to fabricate UV‐crosslinked electrospun fibrous mats of PNIPAM/Magnetite.^66^ In order to obtain a mechanically strong fiber, PNIPAM and Fe_3_O_4_ nanoparticles were mixed with a crosslinking agent of dipentaerythritol hexylacrylate. After electrospinning, fibers of approximately 1 µm in diameter were fabricated. Post‐fabrication fiber mats were then loaded with Vitamin B_12_ by soaking in an aqueous solution of the model drug. Burst release was observed above the LCST value of ∼33 °C. They also observed that subjecting fibrous mat to AMF, also trigger release of therapeutics. Although this construct was utilized for drug delivery, a mechanically strong fibrous mat such as this one has potential for scaffolding or cell sheet engineering applications.

Thermoresponsive hydrogels loaded with magnetic nanoparticles can be magnetically activated to obtain thermal response. A recent study has shown that by adsorbing magnetic nanoparticles on PNIPAM network, LCST of composite network can be modulated.^67^ The authors used this approach for controlled release of entrapped biomolecules, vascular endothelial growth factor, to stimulate cell proliferation. The activity of released growth factor was determined using human umbilical vein endothelial cells. The study showed that bioactivity of released protein was preserved.^67^ In a different approach, magnetic nanoparticles combined with 2D silicates (Laponite® RD), was used for dual‐delivery.^68^ They synthesized magnetic nanoparticles within hydroxypropyl methylcellulose‐*g*‐poly(acrylamide)‐silicate nanocomposites hydrogels. The nanoengineered hydrogels showed response to change in pH as well as temperature. In the pH sensitive study, only a small amount of drug was burst‐released from the initial acidic (pH 1.2) conditions (less than 5% cumulative release), and a burst release of up to 30–50% was found after increasing pH ∼7.4). Incorporation of an alternating magnetic field increased cumulative release to up to 80% under the strongest field. Changes in the AMF demonstrated a direct relationship with the amount of drug released under pH 7.4 conditions.

**Figure 3:**
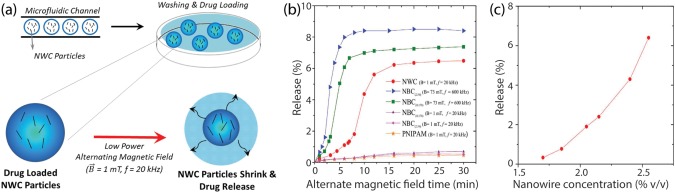
Microfabricated gels loaded with magnetic nanowire. (a) Schematic showing fabrication of magneto‐thermoresponsive NWC particles using microfluidic system. (b) Release of Rh(B) from particles at low‐power and high‐power alternating magnetic fields. NWC show enhanced release at low power filed, when compared to NBC particles. (b) The effect of nanowire concentration can modulate release profile of entrapped molecule. Adapted and reproduced by permission^69^ from Macmillan Publishers Limited © 2016

Traditionally, spherical magnetic nanoparticles are used to fabricate magnetically responsive nanocomposites. A recent study has shown that use of magnetic nanowire can be used to design highly efficient theremoresponsive nanocomposites (Figure 3).^69^ A simple microfluidic technique was used to fabricate PNIPAM microgels loaded with magnetic nanowire in a relatively short time. When subjected to a pulsating magnetic field, microgels loaded with nanowire (NWC), released 70% of entrapped drug with a magnetic field of five orders of magnitude lower power than microgels loaded with superparamagnetic nanobeads (NBC).

## 4 Magnetically triggered thermoresponsive nanoengineered hydrogels for mri applications

Currently, the clinical standard for contrast mediums are gadolinium solutions, however, this heavy ion has a recorded toxicity problem in some patients.^21^ Since greater than 90% of the solution is excreted from the kidneys, gadolinium's potential toxicity makes some physicians reluctant to order frequent MRI scans on patients with kidney problems.^21^ In this section, we review the use of magnetic particles as potential contrast mediums as a way to combat this toxicity issue and still achieve enhanced imaging with contrast mediums. Due to the hysteresis relaxation mechanism, magnetic nanoparticles have been investigated for T2/T2* modalities on MRI scans. The magnetization potential of these particles allows for more intensity and better resolution when combined with diffusion MRI of normal body tissue. Some contrast agents that have been more recently used include magnetic particles, which have special behavior at certain sizes that can cause a secondary response of hyperthermia to surrounding areas.

A nanocomposite that has been commercialized for MRI application in recent years is ferumoxide.^29^ Commercially sold as Feridex® and Endorem®, this MR contrast agent contains SPINs coated with dextran or carboxydextran to form a spherical, nanocomposite colloid of approximately 120–180 nm. These ferumoxides have been shown in clinical trials to increase T2 imaging capabilities by showing an enhanced image from an MRI scan of normal tissue without the construct. Due to high uptake of SPINs in the liver, Feridex® is utilized as a diagnostic tool and a contrast agent to visualize healthy versus cancerous liver tissue. It has been reported that up to 80% of SPIN solutions are taken up into healthy liver tissue by the reticuloendothelial system via physiologically normal Kupffer cells.^34^ Based on this report, this product is most effective for imaging and diagnosis of some liver cancers.

**Figure 4:**
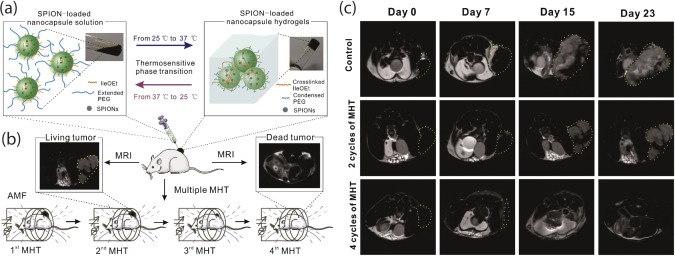
Injectable hydrogels for magnetic hyperthermia treatment. (a) Schematic showing the composition of SPIN‐loaded nanoparticles and thermoresponsive characteristics of nanoparticles results in formation of injectable hydrogels. (b) The nanoengineered system can be used for simultaneous multiple magnetic and MHT and long‐term MRI monitoring. (c) SPIN‐loaded nanoparticles can be used for long‐term MRI monitoring of tumor tissues (marked out with yellow line). Adapted and reproduced by permission^22^ from Elsevier © 2016

Injectable hydrogels are developed for simultaneous MRI imaging and multiple hyperthermia treatment. For example, an injectable nanoparticle‐loaded hydrogel based system was synthesized to facilitate retention of superparamagnetic nanoparticles and allow for multiple cycles of magnetic hyperthermia therapy (MHT) with a single SPIN injection (Figure 4).^22^ A traditional method of tumor treatment, magnetic thermal ablation (MTA) induces tumor cell necrosis at very high temperatures (>50 °C), but some nearby healthy tissue is also damaged through heat conductance. As an alternative to MTA, multiple MHT is proposed, allowing for multiple cycles of heating and cooling at a lower maximum temperature (∼44 °C), to prevent damage to nearby tissues.

**Figure 5:**
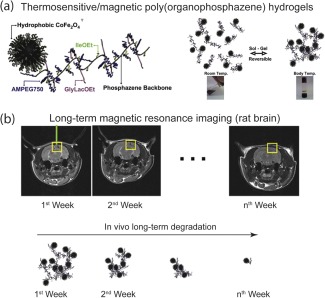
Magnetically responsive hydrogel for MIR imaging. (a) Schematic showing thermosensitive/magnetic poly(organophosphazene) sol‐gel transition. (b) Injected hydrogels used as long‐term MR contrast platform to determine the condition of rat brains. The hydrogel shows in vivo degradation of injected magnetic thermoresponsive hydrogel. Adapted and reproduced^53^ by permission from Elsevier © 2012

Magnetically responsive hydrogels from PPZN and cobalt ferrite (CoFe_2_O_4_) nanoparticles coated with hydrophobic oleic acid and oleylamine were developed for MRI imaging (Figure 5).^53^ In order to have a fully merged solution of CoFe_2_O_4_ and PPZN, hexane was used as the main solvent and then removed via freeze‐drying for in vitro and in vivo experiments. Because the system was formed with a harsh solvent, it was important to test biocompatibility. Cytocompatibility of the hydrogel at various concentrations was tested against mouse and human cell lines and no significant decrease in viability was found in comparison to control after 24 hr. In vivo trials on rats were performed to image a surgically induced puncture in the skull with the developed hydrogel nanocomposite construct. MRI T2 images showed clear contrast present in the skull until 32 days after the stereotactic surgery. Some downsides to clinical applications of this construct include a complex fabrication process and specific storage conditions.

## 5 Magnetically triggered hydrogels for regenerative medicine

**Figure 6:**
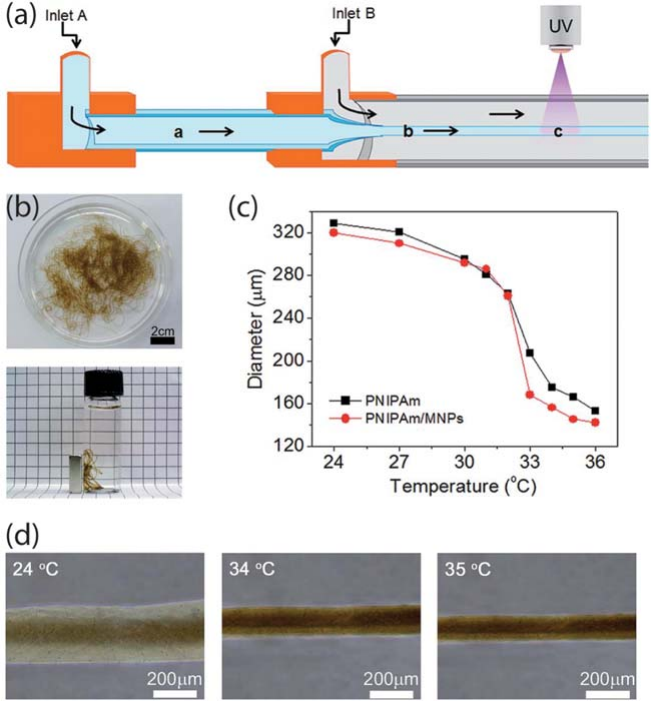
Magnetically responsive microfiber. (a) Schematic showing fabricating of thermoresponsive microfibers loaded with magnetic nanoparticles. Alginate, NIPAM, crosslinker are injected though inlet A, while calcium solution is injected though inlet B to obtain physically crosslinked fiber. UV light was used to obtain (b) chemically crosslinked PNIPAM microfibers. (c) and (d) The diameter of microfiber changes when subjected to different temperature. Adapted and reproduced^9^ by permission from the Royal Society of Chemistry © 2015

Microfabricated structures are used to mimic complex tissue architecture such as a vascular network.^70^, ^71^ In a recent study, stimuli‐responsive microfibers were investigated for remotely controlled cellular response (Figure 6).^9^ Coaxial capillary injection of the PNIPAM/MNP pre‐polymer solution and a calcium alginate template was used to fabricate temperature‐responsive microfibers. The size of the microfibers was controlled by diameter of the microcapillary. Both hollow and solid microfibers can be fabricated by switching the orientation of the calcium alginate with regards to the hydrogel monomer solution. PNIPAM/MNP microfibers showed temperature‐dependent reversible volume transition. It is expected that such stimuli‐responsive microfabricated hydrogels can be used as scaffold for tissue engineering and drug delivery systems. In addition, the pre‐polymer solutions can also be used for 3D bioprinting of complex tissue structures.

In a recent paper, C_10_‐C_20_ saturated fatty acid salts with encapsulated Fe_2_O_3_ nanoparticles were fabricated for potential use in cell sheet engineering and 2D gel constructs.^72^ These fatty acid salts were determined to have phase transition temperatures depending on the carbon chain length. At C_15_ and above, the fatty acid shell was solidified and would only become fluid again once heated. Magnetic forces on the Fe_2_O_3_ nanoparticles allowed both temperature and magnetic fields to control the flow and assembly of these fibers. By utilizing capillary interactions between the fatty acids, magnetic forces forced fiber assembly. When the magnet was turned off, the chains curled in on themselves from full extension along the magnetic axis, but the fibers stayed intact. This dispersion of coated nanoparticles was found to hold its own macrostructure due to nanocapillary bridges from electrostatic forces.

Although PNIPAM is well investigated for engineering cell‐sheets,^6^ magnetically controlled nanocomposites could allow for improved and repeatable controlled removal of cell sheets.^14^ Magnetite can also be utilized for cell sheet engineering. By having cells uptake SPINs, researchers have found that magnetic guidance can help cells to arrange into cell sheets via mechanotaxis.^73^ With potential in both PNIPAM and magnetite, a composite could be fabricated to explore their possibilities for interaction.^45^ Although no cell‐sheet constructs currently have both thermoresponsive and magnetic nanoparticles, and each system creates cell sheets very differently, future research could focus on this combined approach.

## 6 Conclusions

A range of thermoresponsive magnetic hydrogels are developed for on‐demand delivery of biomolecules for a range of biomedical applications, including therapeutic delivery, bioimaging, and regenerative engineering. Although most of the studies are focused on use of spherical magnetic nanoparticles to fabricate smart hydrogels, other shapes and types of magnetic nanoparticles have been developed to enhance the efficacy. In addition, though PNIPAM seems to be the common choice of thermoresponsive polymer, use of other theremoresponsive polymers such as poloxamer and poly(organophosphazene) are also explored. A range of microfabrication approaches to engineer thermoresponsive magnetic microgels are also developed. These fabrication approaches can be used to mimic complex tissue structures.

Development of injectable hydrogels for cell or therapeutic delivery will be highly beneficial for minimally invasive approaches. However, use of these smart systems in 3D bioprinting and other emerging fabrication approaches are not observed. It is expected that injectable magnetic hydrogels can be used for additive manufacturing processes to control and patter cellular behavior. The emergence of bioprinting technologies resulted in the development of 3D printed scaffolds for functional organ engineering, consisting of spatially controlled cell patterns that may be loaded with appropriate biological moieties to control or direct cell fate. The development of thermoresponsive magnetic bioinks will provide significant control over spatially driven design to coordinate cellular arrangements into tissues and organs of interest. By developing stimuli‐responsive bioinks that can respond to external magnetic fields, we can control and direct cellular process to obtain functional and implantable constructs.

## Conflict of interests

There is no financial conflict of interests.
